# Targeting Receptor Kinases in Colorectal Cancer

**DOI:** 10.3390/cancers11040433

**Published:** 2019-03-27

**Authors:** Marilina García-Aranda, Maximino Redondo

**Affiliations:** 1 Research Unit, Hospital Costa del Sol. Autovía A7, km 187. 29603 Marbella, Málaga, Spain; marilina@hcs.es; 2 Red de Investigación en Servicios de Salud en Enfermedades Crónicas (REDISSEC), 28029 Madrid, Spain; 3 Instituto de Investigación Biomédica de Málaga (IBIMA), 29010 Málaga, Spain; 4 Facultad de Medicina, Campus Universitario de Teatinos, Universidad de Málaga, 29010 Málaga, Spain

**Keywords:** colorectal, cancer, kinases, receptor, target

## Abstract

Colorectal cancer is the third most common malignancy in men and the second most common cancer in women. Despite the success of screening programs and the development of adjuvant therapies, the global burden of colorectal cancer is expected to increase by 60% to more than 2.2 million new cases and 1.1 million deaths by 2030. In recent years, a great effort has been made to demonstrate the utility of protein kinase inhibitors for cancer treatment. Considering this heterogeneous disease is defined by mutations that activate different Receptor Tyrosine Kinases (RTKs) and affect downstream components of RTK-activated transduction pathways, in this review we analyze the potential utility of different kinase inhibitors for colorectal cancer treatment.

## 1. Introduction

### 1.1. Colorectal Cancer

Bowel cancer, colon cancer and rectal cancer have many features in common and are often grouped together as colorectal cancer (CRC), the third most common cancer in men and the second most common cancer in women worldwide. CRC represented approximately 10% of all incident cancer cases and caused 8.5% of all cancer deaths in the year 2012 [1]. 

In CRC patients, metastasis is the main cause of cancer-related mortality [2]. Most cases of colon cancer begin as small, benign adenomatous polyps that over time can become malignant. Since during these early stages CRC symptoms may be minimal or non-existent, the prompt reaction of patients to the first symptoms [3,4] and early diagnosis will contribute to treatment success and increase patients’ survival in over 90% of cases [5]. 

However, despite the success of CRC screening programs, approximately 25% of patients present a metastatic disease at time of diagnosis and, during the course of the disease, 40% of them will develop metastases [6] mainly to the liver, lung, the peritoneum or other organs such as the brain, bone, adrenals and spleen [2]. In these patients, the resectability of single-site metastasis will significantly affect patient’s management approach and prognosis [2]. 

Thus, although diagnostic and therapeutic advances have allowed a decrease in CRC incidence rates (6.1% in year 2018), the latest Global Cancer Incidence, Mortality and Prevalence (GLOBOCAN 2018) database still shows a significantly increased mortality rate (9.2%) and an estimate of 1,096,601 new cases and 551,269 deaths worldwide in 2018 [7], which justifies the need to carry on additional studies in the search of new treatment strategies for CRC.

### 1.2. Targeted Therapies for Colorectal Cancer Treatment

Colorectal tumors diagnosed at an early stage are usually successfully handled with first line treatments such as surgery, radiotherapy or conventional chemotherapy. In these patients, 5-year relative survival rate is 88–92% while for patients with stage IIIC this percentage decreases to 58–72% [8]. In recent years, different drug combinations like Folfiri (folinic acid, fluorouracil and irinotecan) and Folfox (folinic acid, fluorouracil and oxaliplatin) have also demonstrated their utility as first-line treatments to improve median survival and progression-free survival of patients with metastatic stage IV. Notwithstanding, 5-year relative survival rate for metastatic CRC remains 12–13% [8] and the search of alternate treatments, a challenge.

Despite the great impact of conventional chemotherapy in cancer treatment, their nonspecific toxicity against rapidly dividing cells and the acquisition of secondary resistances remain a major constraint on achieving optimal results. In this respect, the great progress achieved in the field of molecular oncology has allowed for the development of highly selective treatments designed to induce cancer cell death by interfering with specific genes or proteins involved in cell growth or resistance to apoptosis. Apart from their enhanced tumor selectivity, these highly effective treatments also cause less side effects than conventional treatments, which has turned them into an invaluable tool for cancer treatment.

### 1.3. Molecular Classification of Colorectal Cancer

Molecular profiling techniques including Sanger sequencing, immunohistochemistry (IHC), fluorescence in situ hybridization (FISH), quantitative polymerase chain reaction (qPCR) and Next-Generation Sequencing (NGS) are clinically used to detect alterations at gene sequence level such as DNA mutations, copy number variations and gene fusions across the genome that would be valuable as response biomarkers to different treatment approaches.

Despite the limitations of these rapidly developing technologies, along with bioinformatics they are significantly contributing to improving our understanding of the molecular basis of cancer [9], which is essential for the development of precision medicine designed according to a specific tumor profile. This approach is of great interest since it would allow for the design of specific treatments depending on the alteration present in the tumor regardless of its location.

Molecular profiling has allowed CRC stratification and a better understanding of this heterogeneous and usually lethal disease. Based on independent molecular classification systems, gene expression profiling studies and analytical approaches [10], an international consortium has recently proposed one of the most robust CRC classifications [11]. This novel classification system consists of four Consensus Molecular Subtypes (CMS) (14% CMS1, hypermutated; 37% CMS2, canonical; 13% CMS3, metabolic; 24% CMS4, mesenchymal; and 13% residual unclassified group with mixed features, which is a possible transition phenotype or a reflection of intratumoral heterogeneity) [10] sharing different genetic and molecular changes, including some altered kinases.

On the other hand, the recent molecular pathological classification system proposed by the Cancer Genome Atlas Project is based on a wide-ranging genomic and transcriptomic characterization study using array-based and sequencing technologies [12], which classifies CRC into two major groups: ~16% hypermutated cancers with either microsatellite instability or ultra-mutated cancers with DNA polymerase epsilon proofreading mutations and ~84% non-hypermutated, microsatellite stable cancers with a high frequency of DNA somatic copy number alterations and common mutations in Adenomatous Polyposis Coli (*APC*) and *TP53* tumor suppressor genes, *KRAS* (Kirsten Rat Sarcoma Oncogene 2), *SMAD4* and *PIK3CA* (Phosphatidylinositol 3-kinase, catalytic, alpha).

Both classification systems point out the presence of altered kinases in different CRC subtypes which, despite their novelty, have clinical implications and show great potential as predictive biomarkers for the efficacy of conventional and targeted treatments [13], deserving further research.

### 1.4. Protein Kinases

#### 1.4.1. Protein Kinases as Key Regulators of Cell Function

Protein kinases (PK) catalyze the transfer of phosphate, diphosphate, nucleotidyl residues and other groups to a receptor molecule [14]. The human PK superfamily, which is included in the Enzyme Commission (EC) Class 2.7-Transferring phosphorus-containing groups of the Enzyme List created by the Nomenclature Committee of the International Union of Biochemistry and Molecular Biology [14], is subdivided into 13 subcategories attending to the accepting group. Among these, due to their role as key elements in the regulation of most cellular activities including division, proliferation, differentiation, signal transduction, and apoptosis, proteins belonging to groups 2.7.10 and 2.7.11 (tyrosine-kinases and serine/threonine kinases, respectively) comprise the two major representatives of this large superfamily, having been widely investigated and described in scientific literature.

Protein kinases have also been classically classified based on their location in the cell or in accordance to sequence comparison of their structurally conserved protein domain containing the catalytic core. These classification systems are widely accepted and are commonly used in current scientific literature (Table 1).

The transfer of the gamma phosphate from a nucleotide phosphate, normally an ATP or GTP molecule, to one or more amino-acid residues in the side chain of substrate protein usually results in a conformational change of the targeted protein which affects its function, cellular location or association with other proteins. These reversible reactions, mediated by protein kinases and their agonist phosphatases and often translated as a on or off switch [17], triggers the sequential activation of highly conserved protein kinases ultimately phosphorylating a target protein, and constitutes one of the most prevalent post-translational modifications involved in the regulation of key cellular functions and processes in general (Table 1) and in signal transduction in particular.

Accordingly, over the past decade, many studies have proven the role of protein kinases in human tumorigenesis and cancer progression and have validated their use as targets for cancer treatment [26] (Table 1). As a result of such studies, the tyrosine kinase inhibitor Gleevec (imatinib mesylate) was approved in 2001 for the treatment of chronic myeloid leukemia and its success became a turning point for the development of similar therapeutic approaches to other malignancies, including CRC. At present, different targeted treatment options are available for metastatic CRC patients, including monoclonal antibodies against the vascular endothelial growth factor (VEGF) or the epidermal growth factor receptor (EGFR), either as monotherapy, combined with chemotherapy, or with each other, to enhance patients’ progression-free survival or overall survival [27,28].

#### 1.4.2. Small Molecule Kinase Inhibitors for Cancer Treatment

Apart from genetic mutations, cancer can result from the aberrant functioning of signal transduction pathways and the alteration of normal cell mechanisms controlling gene expression. Given the role of protein kinases and phosphatases as master regulators of cell signaling and the involvement of dysregulated or mutated kinases in human tumorigenesis and cancer progression [8,29], these enzymes have been confirmed as valid candidates for the development of new targeted treatments for cancer treatment.

The designing process of selective small kinase inhibitors is based on the conserved structure and sequence of a kinase catalytic core, in which protein crystal structures, computational molecular modeling and docking studies have been of vital importance [26].

**Figure 1:**
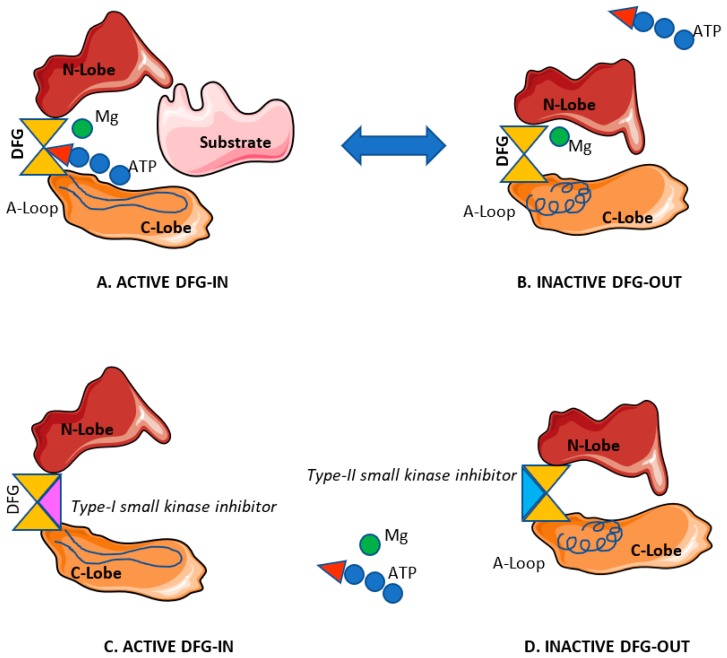
Active and inactive structural fold of model protein kinase. Conformational changes in the catalytic domain is required for kinase function. (**A**) Active protein kinase (open) and substrate molecule. (**B**) Inactive protein kinase (closed) with folded activation loop. (**C**) Type-I small kinase inhibitor competes for the primary ATP-binding domain of kinase active conformation. (**D**) Type-II inhibitors bind to an allosteric pocket adjacent to the ATP-binding site in the inactive state impeding kinase activation. Abbreviations: N-Lobe: N-Terminal subdomain. C-Lobe: C-Terminal subdomain. DFG: (Asp-Phe-Gly)-Motif. Mg: Magnesium. ATP: Adenosine Triphosphate.

Kinase catalytic cores also share a similar structural fold [30], which is characterized by the presence of a smaller N-terminal subdomain (N-lobe) composed of a β-sheet and a long α-helix, a predominantly α-helical large C-terminal subdomain (C-lobe) and an adenosine triphosphate (ATP) binding site in the cleft between them which acts as a hinge [30] during conformational changes. As part of the ATP-binding site, and coordinating magnesium binding, there is a highly conserved Asp-Phe-Gly (DFG) motif immediately followed by a stretch of 20–30 residues known as the activation loop (A-loop), which serves as the regulator of kinase activities [30] (Figure 1).

The structure of multiple protein kinase catalytic subunits has been solved in recent years [31], and has facilitated the development of over a hundred small molecule kinase inhibitors with the ability to modulate protein kinase activity, many of which have already been approved for clinical use in cancer treatment [26].

According to their action mechanism, kinase inhibitors are classified into two main groups: Type-I small kinase inhibitors, which are designed to compete for the primary ATP-binding domain of kinases catalytic core in the active state, and type-II inhibitors, that additionally bind to an allosteric pocket adjacent to the ATP-binding site in the inactive state [30] (Figure 1). 

Although type-II is more selective than type-I inhibitors [32], both type-I and type-II inhibitors impede the phosphorylation of a substrate molecule by the targeted protein kinase and the subsequent inactivation of downstream signal transduction. Since abnormal signaling due to dysregulated kinases can result in uncontrolled cell growth and proliferation, kinase inhibitors can prevent aberrant cell growth or apoptosis inhibition [26,29].

## 2. Altered Kinases in Colorectal Cancer

Advances in the molecular biology of cancer have led to the identification of mutated or dysregulated protein kinases involved in molecular events related to cancer development and progression regardless of tumor origin in the body. These highly relevant findings may have immediate clinical implications, since different protein kinase inhibitors that were firstly designed for the treatment of a different malignancy would be useful for CRC treatment.

### 2.1. Main Altered Kinases in Colorectal Cancer

CRC is a heterogeneous disease defined by mutations that activate different Receptor Tyrosine Kinases (RTKs) and that also affect downstream components of RTK-activated transduction pathways [33].

These alterations have already been reported in CRC molecular classification systems. The Cancer Genome Atlas Network system has described *KRAS* and *PIK3CA* mutations in non-hypermutated CRC tumors [12]. In line with this, the CMS classification reports that hypermutated CMS1 is characterized by MAPK activation while non-hypermutated CRC tumors, englobed into CMS2 subtype, present activating *KRAS* and *PKI3CA* mutations [10]. Notably, although *KRAS* mutants are present in every molecular subtype, they would be more prevalent among CMS3 CRC (68%) [13]. Approximately 3% of CMS3 and 5% of CMS4 CRC also show high copy number for HER2 [13]. To date, the genomic signature of each subgroup has already proved to have clinical implications as a valuable tool to predict patient prognosis and to determine a better treatment strategy for each patient [13].

#### 2.1.1. Receptor Kinases 

RTKs are cell-surface transmembrane receptor kinases composed of an extracellular ligand binding domain, a single transmembrane helix and a cytoplasmic region containing the protein tyrosine kinase activity with C-terminal regulatory regions.

RTK activation leads to autophosphorylation in the tyrosine kinase domain causing a conformational change that allows intracellular ligands docking and the activation of signal transduction pathways [34]. Thus, RTK autophosphorylation is a key initial step in the activation of downstream signaling cascades and accordingly, alterations in their activity, abundance, cellular distribution and/or regulation are present in many types of cancer [34] including CRC (Table 2).

Similar to RTK, Receptor Serine/Threonine Kinases (RSTK) are transmembrane proteins, localized to the plasma membrane, containing extracellular ligand-binding domains and cytoplasmic kinase domains. On the basis of primary amino acid sequence comparison, RSTK are classified into two main subfamilies (Type I and II RSTK), both of which act as signaling receptors for members of the TGF-β superfamily of secreted polypeptides [102]. (Table 3)

Upon ligand binding, a cell-surface complex of type I and type II receptors is formed in which type II receptors phosphorylate the kinase domain of type I partners and allow the binding and phosphorylation of some SMAD proteins [34], which are the main signal transducers for TGF-β receptors and regulate DNA transcription. A third subfamily, type III receptors or accessory proteins, regulate the receptor complex signaling [34]. Due to the important role of TGF-β cytokines in the regulation of cell proliferation, differentiation, adhesion and migration, the dysregulation or aberrant expression of RSTK has a role in different physio-pathological processes including cancer.

Among these receptors, TGFBRs have a great relevance in CRC, since a large proportion of these tumors display mutational inactivation of the TGF-β pathway along with an enhanced TGF-production [105]. Provided the regulatory role of TGF-β in the development of CRC and its metastatic process, different studies have validated this pathway as a valid target for metastatic CRC treatment and have facilitated the development of several strategies targeting TGF-β which are currently in preclinical or clinical trials [106].

#### 2.1.2. Non-Receptor Kinases

Genomic instability together with improved cell survival over time increases the probability that tumor cells will acquire new mutations affecting additional kinases. For this reason, and given the tight interconnection between receptor kinases and down-stream transduction pathways, the effectiveness of receptor-kinase inhibitors as monotherapy would be compromised and would make necessary the use of combination therapies targeting down-stream kinases. 

As in other types of cancer, constitutive activation of central survival pathways involving MAPK or protein kinase B (PKB/AKT) is usually found in CRC cells [107]. Other commonly altered pathways in CRC include *PIK3CA* mutations and *PTEN* (Phosphatase and Tensin homologue deleted on chromosome 10) mutations and deletions which, combined, are found in about 40% of large bowel tumors [108].

##### MAPK Kinases

MAPK kinases (MAPKs) belong to a large family of serine-threonine kinases and constitute the major signaling pathway from the cell surface to pro-survival transcriptional responses within the nucleus including cell growth, proliferation, differentiation, development, transformation, migration or death [26]. The constitutive hyperactivation of MAPKs in the absence of extracellular ligands has a significant role in the pathogenesis, progression, oncogenic behavior [109] and chemoresistance [110] of human CRC.

MAPKs are involved in three major signal transduction pathways [109]:

The MAPK/ERK pathway, also known as the RAS/RAF/MEK/ERK pathway, is located downstream of many growth-factor receptors usually overexpressed or activated in CRC, and upstream several key transcription factors and proto-oncogenes [109]. Indeed, although the mechanisms causing increased MAPK/ERK signaling and enhanced mitogenesis in CRC are multifactorial, one of the main causes is EGFR upregulation [109]. Accordingly, overexpression and constitutive activation of MAPK/ERK pathway has been reported in the carcinogenesis, migration, invasion and metastasis of CRC [35] and its components, as a potentially useful target for CRC treatment [109]. 

Membrane receptor activation triggers RAS (a Small GTPase) phosphorylation and the subsequent consecutive activation of downstream MAP3K (RAF), MAP2K (MEK) and MAPK1 (ERK) kinases. The activation of this enzymatic cascade is of importance in the activation of transcription factors such as Myc. 

The stress-activated protein kinases/c-Jun Nh(2)-terminal kinase (SAPK/JNK) signaling pathway, which can be activated in response to cellular stress and extracellular ligands [111] like cytokines [109], has an important role in the activation of Activating Protein 1 (AP1) [109], a transcription factor regulating gene expression and controlling cellular processes such as differentiation, proliferation and apoptosis [112]. Increased JNK activity has been found in different human malignancies [109].

MAPK14 (p38 MAPK) pathway: despite the anti-proliferative and tumor suppressor activity of MAPK14 in some tissues [110], these kinases have an important role in the regulation of CRC cellular proliferation and differentiation, apparently by activating transcription factors such as STAT1 (signal transducer and activator of transcription factor 1) and protecting cells from apoptosis [109]. Although the MAPK/ERK pathway is a major regulator of cell proliferation in CRC [109], MAPK14 has been identified as an important mediator of resistance to chemotherapy [110]. To date, several MAPK14 inhibitors passed phase I clinical trials and are currently in phase II or III for inflammatory diseases and cancer [110].

Under normal conditions, MAPK pathway is tightly regulated by phosphatases and by bidirectional communication with other pathways, such as PI3K/AKT [113]. 

##### The PI3K/AKT Pathway

The PI3K/AKT cascade is an intracellular signal transduction pathway, involved in apoptosis inhibition and cell growth and proliferation promotion [108], which is altered in 10–15% of CRC [108]. 20% of these alterations are activating missense mutations affecting PI3K gene (*PIK3CA*) [104].

Upon extracellular stimuli (EGF, IGF-1, insulin, CaM, among others), the inactive form of PI3K in resting cells is recruited to the inner surface of the plasma membrane, where its activity is modulated by RAS and SRC family kinases [114]. Activated PI3K phosphorylates AKT, its first bona fide effector in cells [115], which is also located in the plasma membrane. After activation, AKT translocates to the cytoplasm and the nucleus, triggering the phosphorylation of diverse target proteins involved in apoptosis regulation, DNA repair, metabolism, protein synthesis and cell division [114], which finally results in enhanced angiogenesis and epithelial-to-mesenchymal transition [114].

Experimental data and computational simulations have shown that there is a dynamic and complex link between MAPK/RAS and PI3K/AKT pathways at different stages of signal propagation [114]. In short, it seems that MAPK/RAS and PI3K/AKT cross-talk is context dependent and that both pathways can activate or inhibit each other, determining the cell fate [114], since a positive influence of the PI3K pathway on the MAPK pathway would be more effective at low doses of growth factors whereas a negative influence of the MAPK pathway on the PI3K pathway would be mostly pronounced at high doses of growth factors [114]. 

Last but not least, provided that *KRAS* and *PIK3CA* mutations are not mutually exclusive, the constitutive activation at physiological concentration of growth factors of both the MAPK and PI3K/AKT pathways would be a selective advantage [108] for cell survival and cancer progression that would be considered when targeting these pathways.

##### PTEN

PTEN, is a tumor-suppressor phosphatase involved in the inhibition of cellular proliferation, survival and growth by inactivating PI3K-dependent signaling [26]. PTEN is altered in 5–14% of CRC [33,116], resulting in PI3K/AKT signaling upregulation [117] and decreased sensitivity of CRC tumors to anti-EGFR antibodies [33,118,119].

## 3. Targeting Receptor Kinases in Colorectal Cancer

### 3.1. Targeting Receptor Kinases in Colorectal Cancer

Under the selective pressure caused by the immune system or chemotherapeutic agents, deregulated kinases that cause apoptosis inhibition and cell proliferation represent a survival advantage over other cells that, at the same time, implies an addictive effect to kinase signaling and other oncogenic pathways [120,121]. Accordingly, and since CRC is characterized by mutations that activate RTKs, these tumors are highly sensitive to inhibition strategies targeting RTKs.

As the use of single kinase inhibitors has only demonstrated modest clinical benefits [26], over the last years a number of multi-kinase inhibitors have been developed and emerged as a novel strategy with a greater potential than existing humanized monoclonal antibodies (mAbs) such as anti-HER2 mAb (trastuzumab) or anti-VEGF mAb (bevacizumab) [54]. Many of these kinase inhibitors, which have already been approved by the FDA, would also be valuable for CRC treatment since they have demonstrated their efficacy in blocking RTKs that are frequently altered in CRC. (Table 4).

However, despite the encouraging results that multi-kinase inhibitors have achieved in the treatment of different types of cancer, preclinical studies in the field of CRC are showing contradictory results and just one multi-kinase inhibitor, Regorafenib, has been approved for advanced CRC treatment and other malignancies with a common altered kinase [144].

In this regard, FDA approval of pembrolizumab (Merck & Co. Inc., Keytruda, Kenilworth, NJ, USA) in 2017 as the first drug for the treatment of adult and pediatric patients with a common biomarker [145], has been a decisive milestone in current cancer therapeutics. The first approval of drug which has been designed on the basis of tumor genetics rather than tissue type or tumor site, has facilitated larotrectinib (Vitrakvi, Loxo Oncology Inc. and Bayer) FDA accelerated approval on November 2018 of for adult and pediatric patients with any advanced solid tumor with *NTRK* gene fusion without a known acquired resistance mutation [55]. Larotrectinib approval has represented the second tumor-agnostic cancer treatment approved by the FDA and hopefully the beginning of a new era in which drugs are designed to modify disrupted pathways in cancer cells and therefore, valid for the treatment of different malignancies including CRC, which will be reflected in the design of clinical trials and, therefore, in a faster release of new therapeutic options.

### 3.2. Overcoming Resistance to Kinase Inhibitors

Despite the promising prospects of kinase inhibition in CRC, results of preclinical and clinical studies are not always proving as satisfactory as expected. Indeed, less than 20% of patients with metastatic CRC respond to clinically available targeted drugs when used as monotherapy [33], justifying the search of new therapeutic approaches. 

Studies carried out in this regard show that, given the existence of complex cross-talks among different kinase cascades, targeting one pathway would cause an imbalance between interacting kinases and the activation of compensatory signaling resulting in apoptosis evasion [109]. For this reason, and according to different studies in this regard, combined inhibition of different altered kinases would be more efficient inhibiting cancer cells growth and viability than targeting the components of each pathway alone [26,114,146]. However, the applicability of combinations of different kinase inhibitors with standard treatment or immunotherapy would be significantly limited by accumulating toxicities and side effects and justifies additional studies. 

On the other hand, given the genomic instability of cancer cells, there is a high probability that the receptor kinase is altered by mutations and causes resistance to selective inhibitors [34], which would require a prior study of patient’s personal genomic profile in order to select the most appropriate treatment. At the same time, genomic instability can cause the alteration of different kinases within the cell or within the same transduction pathway, which would result in ineffectiveness or partial response to the targeted treatment. This phenomenon has been widely studied in CRC cells resistant to anti-EGFR treatment.

The aim of EGFR-mAbs is to prevent the activation of down-stream transduction pathways, such as MAPK/RAS and PI3K/AKT, as key components of cell proliferation and survival in EGFR-dependent cells [33]. For this reason, EGFR expression was initially used as biomarker for anti-EGFR treatment [35]. However, given the low response of patients, it was later found that the value of EGFR-expression as a biomarker was insufficient. 

Since oncogenic mutations in genes encoding key downstream effectors within EGFR-signaling pathway are responsible for primary intrinsic resistance and reduced efficacy of EGFR-mAbs [33], further understanding of key components of the EGFR-signaling pathway that are frequently altered in metastatic CRC has resulted in a significant step towards improving patient selection and outcome. Among these altered components, the one that has been best studied to date is RAS.

RAS is an important effector of EGFR signaling mainly, but not exclusively, through RAF, MAPK and also PI3K pathways [33]. *KRAS* proto-oncogene, which is mutated in approximately 32–40% of CRC tumors [33,109], has been described as an early event in the development of CRC [109] in which the GTPase activity of KRAS is disabled and downstream signaling is inoperative. For these reasons, mutant *KRAS* has become a predictor of resistance to EGFR-mAbs and has allowed for the implementation of personalized medicine in CRC patients according to their genotype (*KRAS*-wild or mutated) [33,147]. In such manner, the RAF-coding gene, *BRAF*, which is also found to be mutated in 9–15% [33,109] of CRC and usually associated with poor prognosis [33], limited response to standard-of-care therapies [148], and with an increased kinase activity in sporadic CRC with microsatellite instability [109], would also be a valuable biomarker. However, and in contrast to *KRAS* mutations, there is still insufficient evidence to demonstrate the value of *BRAF* mutations as predictive biomarker of survival benefit from anti-EGFR mAbs in CRC [148]. 

The ineffectiveness of EGFR-antibodies in CRC patients with mutations in the *KRAS*, *NRAS* or *BRAF* genes [149], requires the search of alternate treatments such as the use of DDR1 inhibitors like Nilotinib, which has demonstrated to be effective to inhibit the invasive and metastatic behavior of CRC through a RAS-independent mechanism [92].

Within this context, a throughout preliminary study of patients along with the search of predictive biomarkers based in additional molecular alterations in CRC patients that could affect the efficacy of kinase inhibitors have signaled the emergence of a new era in which patients that would benefit from different kinase inhibitors are previously selected.

## 4. Conclusions

Protein kinase inhibitors have emerged as a promising therapeutic option for the treatment of cancer patients including those with advanced CRC. However, the complex cross-talks among different kinase cascades along with the existing heterogeneity at the kinome level and the high probability of mutations, are the cause of resistance to selective inhibitors and have proved to be a major impediment to achieving the desired results. Although there is still the need of further research to better understand the complexity of kinases pathways and their role in CRC in order to design rational combination therapies that allow enhanced response and minimal resistance to treatment, numerous studies have already demonstrated the effectiveness of combination therapies and multi-kinase inhibitors for cancer treatment in both in vitro and in vivo.

Although the currently available options for CRC patients are still limited, the great advances achieved during the last decade in the field of molecular oncology have become a turning point by making it possible to treat patients with different tumors but with a common gene mutation affecting a receptor kinase. Given the importance of receptor kinases in CRC tumorigenesis and progression, this new approach will serve to considerably enhance the number of therapeutic options for progressing CRC patients in the near future.

Nevertheless, there is still a need to investigate which kinases would be valuable as predictive biomarkers as well as their role in CRC so that new kinase inhibitors could be developed in the future. Finally, it is worth highlighting the potential of CRC stratification systems based on CRC molecular profiling as promising tools to predict patient response, implementation of CRC subtype-based interventions or clinical trial design.

All of this leads us to personalized cancer medicine based on genetic profiling of tumors that targets an individual’s unique mutational profile in which a preliminary study and selection of CRC patients that would better benefit from each combination treatment would be required.

## Figures and Tables

**Table 1:** Human protein kinases classification based on cell location and catalytic core sequence comparison.

Criteria	Overview		Role and Significance
Location in the cell	Transmembrane Receptor Kinases	Consist of a ligand-binding extracellular domain and a catalytic intracellular kinase domain.	Key initial step in the translation of an extracellular stimulus as down-stream signaling cascades within the cell.
	Non-receptor Kinases	Lack transmembrane domains. Located in the cytosol, nucleus or associated to the inner surface of plasma membrane.	Signal transduction throughout the cytoplasm and the nucleus and gene transcription.
Eukaryotic catalytic domain sequence	AGC	Subgroup of 60 Serine/Threonine kinases, including A, G and C protein kinases (PKA, PKG and PKC respectively), all of them with high homology of the catalytic kinase domain [15]. This subfamily also includes well-studied enzymes such as AKT (PKB), S6K, RSK, MSK, PDK1 and GRK as well as SGK, NDR, LATS, CRIK, SGK494, PRKX, PRKY and MAST [15].	Most AGC kinases are activated by phosphorylation [15]. AGC kinases are involved in numerous cellular processes including metabolism control, protein synthesis (AKT, RSK), cell proliferation (AKT), mediation of growth factors and hormones effect (PKC, PKA) or sodium transport (SGK) [15] and their mutation and/or dysregulation has been related to multiple human diseases including cancer, diabetes and different inherited syndromes [15].
	CAMK	The Ca^2+^/Calmodulin-dependent protein kinases (CAMK I, CAMK II, CAMK III, CAMK IV, CAMK V) are Serine/Threonine kinases with a highly conserved architecture of their active pocket which contains a bi-lobed catalytic domain followed by a regulatory domain with both an autoinhibitory and a CaM-binding domain [16]. CAMK kinases are activated in response to an increase in the concentration of intracellular calcium ions [17].	CAMK, whose functionality are regulated by Ca^2+^-binding protein calmodulin (CaM), play a key role in processes such as gene transcription, apoptosis or cytoskeletal reorganization [16] and are responsible for the phosphorylation of various transcription factors [17]. CAMPK dysregulation has been related to cancer progression and therapy response [18].
	CK1	Members of the Casein Kinase (CK) 1 family (alpha, beta 1, gamma 1, gamma 2, gamma 3, delta, epsilon) are monomeric Serine/Threonine kinases with highly conserved regions within the kinase domain but differing in length and sequence of the N-terminal and the C-terminal non-catalytic domain, the last with a crucial role in substrate specificity and in the regulation of kinase activity [19].	Members of the CK1 family act as regulators of signal transduction pathways [17], playing a key role in the phosphorylation of regulatory molecules involved in DNA-transcription and repair, cell proliferation, cytoskeleton dynamics, vesicular trafficking, apoptosis and cell differentiation [19] among others. CK1 are also able to alter the activity of key proteins involved in signal transduction and signal integration molecules [19]. Aberrant expression of CK1 is usually detected in different malignancies including kidney, breast, pancreas and ovarian cancer [19].
	CMGC	CMGC kinase family is named after the initials of its subfamily members: CDK (cyclin-dependent kinases), MAPK (mitogen activated protein kinases), GSK3 (glycose synthase kinase-3) and CLK (cdc2-like kinases). CMGC enzymes are Serine/Threonine kinases which preferentially phosphorylate substrates with proline at the P+1 position [20]. CMGC family members are also characterized by a unique regulatory mechanism that involves a phosphorylated tyrosine in the activation loop or a pre-phosphorylated residue in the substrate [20].	CMGC family members are involved in the regulation of cell cycle (CDK), signal transduction, cell proliferation, differentiation and death (MAPK), glycogen metabolism and embryonic development (GSK3), gene transcription (CLK) [17]. CMGC dysregulations have been linked to oncogenic transformation [17].
	RGC	Members of the Receptor Guanylate Cyclase (RGC) subfamily have an N-terminal extracellular ligand binding domain, a single-pass transmembrane domain and a C-terminal intracellular domain [21] which catalyzes the synthesis of cyclic guanosine monophosphate (cGMP) from GTP. RGC intracellular domain contains a region with sequences homologous to protein kinase core [21], being usually classified as pseudo-kinases. The kinase domain can bind to ATP, causing a conformational change which is thought to regulate the guanylate cyclase domain [22]. RGC kinases can be activated by hormones, peptides and low calcium-induced guanylyl cyclase-activating proteins [23].	RGC kinases play a key role as transducers of extracellular information to the interior of the cell, since intracellular cGMP is a second messenger that modulates the activity of different intracellular protein kinases.
	STE	Based on their homology to the yeast proteins [24], the homologues of yeast Sterile (STE) kinase group is classified into three main families (Ste7, Ste11 and Ste20) which sequentially activate each other to then activate the MAPK family [17].	Members of the STE family are critical regulators of multiple signaling pathways and their aberrant expression is found in different malignancies [24].
	TK	The Tyrosine Kinase (TK) group includes receptor and non-receptor (cytosolic) kinases [24] that specifically phosphorylate tyrosine residues [17]. This subfamily includes the human epidermal growth factor receptor (HER/EGFR) family, the insulin receptor (IR), the insulin-like growth factor 1-receptor (IGF1-R), the SRC, ABL and JAK kinases [24].	TKs are important mediators in transmembrane signaling and signal transduction within the cell, being involved in cell proliferation, differentiation, migration, metabolism and apoptosis in response to internal and external stimuli [25]. Since multiple studies have identified TKs dysregulation during the pathophysiology of cancer [25], decreased apoptosis and increased cell proliferation, this group contains the majority of targets for kinase inhibitors that are currently in clinical use [24].
	TKL	Tyrosine kinase-like (TKL) protein kinases are mostly serine/threonine kinases [24] with sequence similarity to TKs but lacking TK-specific motifs. This group, which contains both receptor and non-receptor kinases, includes the RAF (Rapidly Accelerated Fibrosarcoma) kinases and the transforming growth factor beta (TGF-β) receptors.	Members of the TKL family are involved in the MAPK pathways (RAF/MAPK), in cellular processes such as cell growth, differentiation and apoptosis (TGF-β) and angiogenesis and vascular development (TGF-β-I receptor activin receptor-like kinase, ALK1).

**Table 2:** Receptor Tyrosine Kinase (RTK) Subfamilies classification based on kinase domain sequence and their role in colorectal cancer.

RTK		Overview	Role in CRC
I	Epidermal Growth Factor (EGF/ErbB) receptor family: EGFR, HER2, HER3, HER4 receptors	EGFR can respond to and be activated by protein hormones, cytokines or growth factors, acting as key regulators of decisive cellular processes such as proliferation, differentiation, survival, metabolism, migration and cell cycle control [34].	Positive EGFR expression is a significant independent negative prognostic factor for CRC disease-free survival and overall survival [35]. Positive EGFR expression is also significantly associated with tumor-node-metastasis (TNM) stage T, with a predictive value for postoperative relapse in these patients [35]. EGFR is overexpressed in up to 97% of CRC patients and significantly associated with highly malignant behavior [35,36]. Indeed, as in the case of other malignancies, including breast or lung cancer, EGFR plays a crucial role in the tumorigenesis and tumor progression of CRC and has become a valuable target in the treatment of metastatic CRC.
II	Insulin Growth Factor/Insulin receptor family (IGFR/InsR): IGFR and IRR receptors	Both IGF1 and IGF2 bind and activate IGF1R transmembrane receptor kinase. IGF2R does not contain a kinase domain and binding with IGF2 does not result in downstream signaling [37]. IGFR responses, which include apoptosis and autophagy inhibition, DNA synthesis or amino acid uptake [38], are mediated through intracellular adaptor proteins [34].	The InsR/IGF1R have a major role in the pathogenesis and progression of CRC, contributing to the transformation of normal colon epithelial cells and the development of resistance to both chemotherapeutic drugs and epidermal growth factor receptor targeted agents [37].
III	Platelet Derived Growth Factor Receptor (PDGFR), Colony stimulating factor 1 receptor (CSF-1R) (Ems), KIT proto-oncogene receptor tyrosine kinase (KIT) and FMS related tyrosine kinase 3 (FLT3) receptors	PDGFs are important growth factors for normal tissue growth and division with a role in blood vessel formation [39]. Cancer cells can escape immune responses by secreting CSF to the tumor environment, which stimulates the proliferation and recruitment of immunosuppressive myeloid cells [40]. Accordingly, intratumoral presence of myeloid cells expressing CSFR correlates with poor survival in different malignancies [41]. In CRC, KIT activation by Stem Cell Factor (SCF) ligand induces signaling by different pathways including PI3K, RAS and JAK/STAT [42].	PDFGs are often over-expressed or mutated in CRC stromal cells, pericytes and CRC cell lines [43]. In CRC, PDGF overexpression is associated with angiogenesis, invasion, metastasis, poor survival and resistance to targeted therapies in CRC patients [39], having been proposed as useful biomarker for both diagnosis and CRC treatment [39]. CF1R dependency by intestinal macrophages along with CSF1R overexpression in CRC tumors correlates with tumor stage and differentiation [44]. KIT mutations are usually found in different malignancies including CRC and associated with resistance to chemotherapy and malignant mesothelioma [45]. FLT3 amplification has been reported in approximately 3% of CRC samples associated with primary or acquired resistance to EGFR blockade [46]. Binding of TLT3 ligand to FLT3 triggers PI3K and RAS pathways, leading to increased cell proliferation and apoptosis inhibition [46].
IV	Vascular Endothelial Growth Factor (VEGF) receptor family: VEGFR-1, VEGFR-2, VEGFR-3 receptors	Key regulators of metabolic homeostasis, cell proliferation, migration, tubulogenesis [47], angiogenesis and lymphangiogenesis [34].	Due to its role in regulating endothelial cells differentiation, VEGFR2 is one of the major angiogenesis mediators in CRC [48]. VEGFR2 overexpression correlates with differentiation, metastasis, recurrence and poor prognosis of CRC patients [49]. Interestingly, it has been reported that CRC patients with good overall survival and/or good metastasis free survival are characterized by low VEGFR1 and high VEGFR2 expression [48]. Likewise, VEGFR3 is usually found to be overexpressed in CRC tumor vasculature [50].
V	Fibroblast Growth Factor (FGF) receptor family: FGFR1, FGFR2, FGFR3, FGFR4 receptors	Mediate progenitor cells growth, differentiation, survival and patterning during embryonic development and organogenesis as well as metabolic functions, tissue repair and regeneration in adult tissues [51].	All four FGFR and their ligands are expressed in CRC [52]. Among them, FGR1 is usually overexpressed in CRC patients, correlating with an aggressive clinical behavior [53]. FGFR2 regulates CRC cells migration, invasion and growth and plays an important role in cancer progression [52].
VI	Protein tyrosine kinase-like 7 (PTK7)/ Colon Carcinoma Kinase 4 (CCK4) receptor	These receptors are associated with epithelial cells polarization and neural structures development [34]. Although sequence analysis suggests that the gene product is catalytically inactive as a protein kinase, it is involved in Wnt [34] and VEGF signaling [54].	PTK7, which can promote survival, motility and invasion of cancer cells through non-canonical Wnt-signaling activation [54], is usually overexpressed in colon cancer [54]. However, as a result of complexity in the canonical and non-canonical Wnt signaling network [54], PTK7 upregulation can result in tumor promotion or suppression in a cell context-dependent manner [54], resulting in both favorable o poor prognosis in patients with CRC patients [54]. For these reasons, PTK7-targeted treatments might only be beneficial for CRC patients with oncogenic PTK7 upregulation [54].
VII	Neurotrophin receptor/Tropomyosin Receptor Kinase (TRK, NTRK) family: TRKA, TRKB, TRKC receptors	TRKA, TRKB and TRKC receptors respond to Nerve Growth Factor (NGF), Brain-derived Neurotrophic Factor (BDNF) and Neurtrophin-3, respectively [34] and mediate proliferative and migration processes in neural systems [34].	Food and Drug Administration (FDA) has recently approved larotrectinib (Vitrakvi) for the treatment of patients with solid tumors affected by *NTRK* gene fusions [55]. In CRC patients, chromosomal rearrangements involving the *NTRK1* gene (encoding the TRKA protein) are shown in a small subset of patients, associated with the constitutive activation of the TRKA kinase domain as well as with proliferation and survival in CRC tumors [56]. 16% of samples from CRC patients present *NTRK* gene rearrangements [57] along with high microsatellite instability [57], suggesting that these patients may benefit from both tyrosine kinase inhibitors and checkpoint inhibitors as either monotherapy or in combination [57].
VIII	Receptor Tyrosine Kinase-like Orphan Receptors (ROR) family: ROR1 and ROR2 receptors	Act as alternative receptors and coreceptors of Wnt signals [58], regulating cell proliferation and polarity as well as tissue maintenance.	ROR1 is usually overexpressed in CRC cells when compared to the adjacent normal tissues and positively associated with the clinical stage and lymph-node metastasis, having been proposed as a novel prognostic marker and therapeutic target for CRC [59]. As non-canonical Wnt signaling mediator, ROR2 has a dual role as tumor suppressor or activator depending on tumor type [60] or stage. In CRC, ROR2 overexpression correlates to decreased tumor size [60] is frequently epigenetically inactivated by promoter hypermethylation in the early stages, contributing to CRC progression [61].
IX	Muscle-Specific Kinase (MuSK) receptor	Associated with the formation and organization of the neuromuscular junction from the skeletal muscle side [34].	MuSK receptor is usually expressed in rectum and colon tissues and has been proposed as a potential drug target in CRC [62].
X	Hepatocyte Growth Factor Receptor (HGF) receptor family: mesenchymal-epithelial transition factor (MET) and (Recepteur d’Origine Nantais) RON receptors	HGF stimulates proliferation, migration and morphogenesis of epithelial cells by binding to and activating its receptor c-Met (MET) [63,64].	Genomic instability causes *HGF* gene activation in colon cancer cells, promoting their resistance to necroptosis [65]. Since HGF induces proliferation, motility, adhesion and invasion of CRC cells [66] and is related to CRC development, progression and metastasis [35] high levels of HGF have been proposed as a valuable prognosis biomarker in CRC [35] as well as a marker of tumors with aggressive biology [67,68]. In CRC, high levels of HGF are usually accompanied by the overexpression of c-MET receptor, which is associated with CRC invasion and distant metastases [69] due to c-Met activation of different proteins like survivin, livin and X-linked inhibitor of apoptosis protein (XIAP), which inhibit apoptosis proteins (IAP), through AKT pathway [66]. In this regard, HGF has been proposed to protect CRC cells against EGFR inhibition via c-MET activation [64] and also against glucose starvation-induced apoptosis, promoting resistance to both anti-EGFR agents [64], anti-glycolytic agents and angiogenesis inhibitors [70]. Provided that RON kinase, which is overexpressed in 60% of human colon cancers [71] and altered in certain primary colon cancers, has been related to CRC progression and metastasis [72,73,74], it has recently been proposed as a novel target for advanced CRC patients [71].
XI	TAM (TYRO3-, AXL- and MER-TK) receptor family: AXL, TYRO3, MERTK receptors	TAM receptors can be activated by the vitamin K-dependent proteins Growth arrest specific protein 6 (Gas6) and protein S, affecting cell proliferation, survival, adhesion and migration [75]. TAM act as potent inhibitors of inflammation and have an oncogenic role in a number of cancers [76].	AXL tyrosine kinase receptor is overexpressed in CRC [77,78], having a role in epithelial to mesenchymal transition, tumor angiogenesis, resistance to chemotherapy and targeted agents and decreased antitumor immune response [78]. AXL has also been proposed as a negative prognostic biomarker for CRC patients [78] and as a predictive biomarker of lack of efficacy in RAS-wildtype metastatic CRC patients treated with chemotherapy and cetuximab [79]. As a result, AXL has been proposed as a novel therapeutic target for CRC treatment [77], in particular in those cases in which the adjuvant disease in which EGFR/VEGF-targeted therapies have failed [80]. Apart from AXL, TYRO3 and MER have also been proposed as potential targets in CRC [81].
XII	Tyrosine Kinase with Immunoglobulin-like and EGF-like domains (TIE) or angiopoietin receptor family: TIE1 and TIE2 receptors	Modulators of angiogenic and lymphangiogenic responses [82].	Angiopoietin 2 (Ang-2), TIE2 and VEGFR2 are involved in the development, invasion, angiogenesis, metastasis and prognosis of CRC [83]. In this regard, TIE2 expression has been validated as tumor vascular response biomarker for VEGF inhibitors in metastatic CRC [84] Provided the relation between Ang-2 and TIE2 expression, the Ang/TIE2 signaling pathway has been proposed to have an important role in the progression of CRC [85].
XIII	Ephrin (Eph) receptor family: EphA1, EphA2, EphA3, EphA4, EphA5, EphA6, EphA7, EphA8, EphA10, EphB1, EphB2, EphB3, EphB4, EphB6 receptors	Implicated in the regulation of neuronal development, cell migration, patterning and angiogenesis [34].	The role of the different members of Eph family, the largest one of RTK, is complex. In the early stages (I/II) of CRC malignant transformation, EphA1, EphA2, EphB1, EphB2 and EphB4 are upregulated and may play a role in tumor migration/invasion and metastatic behavior [86]. During CRC progression, Eph expression is gradually reduced until the loss of Eph expression in late stage CRC, which has been proposed as a potential valuable marker for these patients [86].
XIV	Rearranged During Transfection (RET) receptor	After activation by glial cell derived neurotrophic factor family ligands, RET receptors mediate a wide range of responses such as cell proliferation, neuronal navigation, cell migration and cell differentiation [87].	RET has been proposed as a tumor suppressor kinase in CRC [88]. RET inactivation, due to *RET* gene aberrant methylation or mutations, would be involved in the progression of colon adenomas to cancer [88,89]. Provided that rearrangements affecting *RET* is present in 22% of samples from CRC patients [57] along with high microsatellite instability [57], these patients may benefit from both tyrosine kinase inhibitors and checkpoint inhibitors as either monotherapy or in combination [57].
XV	Related to Tyrosine Kinase (RYK) receptor	RYK contains functional extracellular Wnt-binding domains and is implicated in Wnt signaling [90].	RYK role in CRC is under study.
XVI	Discoidin Domain Receptor Family (DDR) receptor family: DDR1, DDR2 receptors	DDR1 is activated by collagen, one of the major components of the extracellular matrix. After activation, DDR1 modulates cell adhesion, proliferation and metalloprotease expression [91].	In a collagen rich environment, DDR1 can promote tumor cell invasion and cancer stem cell survival [92]. DDR1 has a role in invasive and metastatic abilities of CRC cells [92]. Interestingly, *KRAS* mutations induce DDR1 expression and sustains Notch oncogenic signaling and tumorigenesis [92].
XVII	Reactive Oxygen Species (ROS) receptor family	Although ROS ligand and normal function have not been fully identified yet, aberrant expression of ROS has been reported in different malignancies [93], which has turned this protein in a potential target for anticancer drugs.	Genomic fusions causing ROS1 kinase constitutive activation and uncontrolled cellular proliferation are observed in CRC, which has been proposed as a potential therapeutic target in CRC [94,95].
XVIII	Lemur receptor kinases (LMR/LMTK): 1, 2, 3	The precise role of these receptors has not yet been defined [34].	LMTK3 expression is significantly correlated with lymph node metastasis and overall survival in CRC patients, having been proposed as a prognostic marker for these patients [96].
XIX	Leukocyte Tyrosine Kinase (LTK) receptor family: LTK (Leucocyte Receptor Tyrosine Kinase) and ALK (Anaplastic Lymphoma Kinase)	LTK endogenous ligands and precise roles are unknown [34]. Studies with chimeric proteins have shown LTK ability to promote growth and cell survival [97]. Genomic fusions causing ALK constitutive activation and uncontrolled cell proliferation are usually found in human cancer [98].	Only a few cases of CRC show moderate immune-staining for TLK [99]. Genomic fusions affecting *ALK* are observed in CRC, which has been proposed as a potential therapeutic target in CRC [94,95].
XX	Serine/threonine/tyrosine kinase (STYK) receptor: STYK1	Involved in different cellular and developmental processes such as cell proliferation, differentiation and survival [100].	STYK1 overexpression may be involved in the progression of CRC [101]. Aberrant expression of STYK1 has been reported in colorectal cancer with a prognostic value [101].

**Table 3:** Receptor Serine/Threonine Kinase Subfamilies [34].

RSTK	Description	Overview
Type I	Activin Receptor-like kinases (ACVR/ALKs): Activin A Receptor Type 1L (ACVR1L, ALK1), Activin A Receptor Type 1 (ACVR1, ALK2), Bone Morphogenetic Protein Receptor Type IA (BMPR1A), Activin A Receptor Type 1B (ACVR1B, ALK4), Transforming Growth Factor β Receptor 1 (TGFBR1), Bone Morphogenetic Protein Receptor Type IB (BMPR1B), Activin A Receptor Type 1C (ACVR1C, ALK7).	Since TGF-β signaling reduces proliferation and promotes apoptosis and differentiation in colon epithelial cells, loss of TGF-β signaling is considered a feature of CRC cells [103]. *ALK* gene is rearranged, mutated, or amplified in different tumors [100]. In the particular case of CRC, *ALK* fusions are frequent [104]. Alterations affecting the expression and activity of TGF-β receptors and SMAD protein signal transducers determine if proliferation of CRC cell is inhibited [103].
Type II	Activin A Receptor Type 2A (ACVR2A, ActR2), Activin A Receptor type 2B (ACVR2B, ActR2B), Anti-Mullerian Hormone Receptor type 2 (AMHR2, MISR2), Bone Morphogenetic Protein Receptor Type 2 (BMPR2), Transforming Growth Factor Beta Receptor 2 (TGFBR2)	Under study
Type III	Transforming Growth Factor Beta Receptor 3 (TGFBR3)	Under study

**Table 4:** Receptor Kinase Inhibition in CRC.

Targeted RTK	Knockdown Effect on CRC Cells	Current Status for CRC Patients	FDA Approved Multi-Kinase Inhibitors
EGFR	Significantly reduces CRC cell proliferation, colony formation and migration [122].	FDA approved Cetuximab (Erbitux) and panitumumab (Vectibix) monoclonal antibodies for the treatment of patients with EGFR-expressing, metastatic colorectal carcinoma [123,124].	Afatinib, Brigatinib, Dacomitinib, Dasatinib, Erlotinib, Gefitinib, Lapatinib, Osimertinib, Vandetanib.
IGF1R/IGF1-IGF2	IGF1R knockdown inhibits human CRC cell growth and downstream PI3K/AKT pathway [37].	Antibodies targeting IGF1, IGF2 and the extracellular portion of the IGF1R receptor are in clinical trials [34]. Despite the promising results of preclinical and clinical studies, phase II and III trials are showing disappointing conclusions, justifying additional studies for the validation of predictive biomarkers in CRC patients [37].	Brigatinib, Ceritinib
PDGFR	Reduction in cell growth, proliferation an invasion [125].	Multikinase inhibitor regorafenib FDA approved for the treatment of patients with metastatic CRC whose disease has progressed after prior therapy [126]. Crenolanib, a kinase inhibitor in development for the treatment of multiple malignancies, is under clinical trial for the treatment of patients with advanced gastrointestinal stromal tumors with *PDGFRA* mutations [127].	Axitinib, Dasatinib, Imatinb, Lenvatinib, Nilotinib, Nintedanib, Pazopanib, Ponatinib, Sorafenib, Sunitinib
CSFR	Reduces intestinal macrophages in CRC patients, reducing epithelial-to-mesenchymal transition and matrix remodeling [41].	To date, different CSF1 inhibitors are in clinical development both as monotherapy or in combination with conventional treatment or immunotherapy [41].	Sunitinib
KIT	Decreases tumor growth and colony forming capacity [42].	Different preclinical studies with KIT inhibitors are showing encouraging results for CRC prevention and treatment [128]. FDA approved multikinase inhibitor regorafenib for the treatment of patients with metastatic CRC whose disease has progressed after prior therapy [126].	Cabozantinib, Dasatinib, Imatinib, Lenvatinib, Pazopanib, Ponatinib, Sorafenib, Sunitinib
FLT3	Under study	FLT3 amplification in CRC seems to be a passenger alteration that occurs as a late event and might not be the most effective alteration for therapy [46].	Brigatinib, Cabozantinib, Gilteritinib, Midostaurin, Nintedanib, Ponatinib, Sorafenib, Sunitinib
VEGFR	VEGFR1 inhibition decreases tumor growth and metastasis [129].	Bevacizumab and Ramucirumab FDA approved for the treatment of locally advanced or metastatic gastric cancer [129,130]. Multikinase inhibitor regorafenib FDA approved for the treatment of patients with metastatic CRC whose disease has progressed after prior therapy [126].	Axitinib, Cabozantinib, Nintedanib, Pazopanib, Ponatinib, Regorafenib, Sorafenib, Lenvatinib, Vandetanib
FGFR/FGF	Inhibits cell proliferation and tumor growth and enhances tumor cell sensitivity to chemotherapy [131]	Multikinase inhibitor regorafenib approved for the treatment of patients with metastatic CRC whose disease has progressed after prior therapy [126].	Lenvatinib, Nintedanib, Pazopanib, Ponatinib
PTK7	Decreases cell proliferation, drug-resistance and cell migration [132].	PTK7 expression has been proposed as prognostic and predictive biomarker [132] and different PTK7-targeting agents are under development [54].	Under development
TRK	Inactivation of TRKA and down-regulation of downstream signaling pathways followed along with cell proliferation inhibition [133].	Entrectinib, Larotrectinib [56] and Milciclib has shown promising clinical responses in patients with colon cancer and are under clinical trial in patients with other different malignancies [134]	Cabozantinib, Larotrectinib, Milciclib
ROR	As non-canonical Wnt signaling mediator, ROR2 has a dual role as tumor suppressor or activator depending on tumor type [60] or stage.	Before ROR-selective inhibitors can truly be used as valuable targets in CRC a better understanding of Wnt signaling pathways in human carcinogenesis is needed.	Under study
HGFR/c-Met	Prevents distant recurrence of rectal cancer after preoperative chemoradiotherapy [135]. In combination with glucose metabolism inhibition, enhances the effect of angiogenesis inhibitors in CRC treatment [70].	Different clinical trials have evaluated *MET* inhibitors alone or in combination with cytotoxic chemotherapy in patients with gastrointestinal cancer, most of them showing no efficacy [69]	Crizotinib
AXL	There is strong evidence for the potential utility of AXL inhibitors to decrease the metastatic potential of CRC as well as to overcome resistance to immune checkpoint inhibitors, conventional chemotherapy and targeted therapies [78].	AXL represents a promising tool for CRC treatment. Evidence of this is the growing number of AXL inhibitors that are being developed and the ongoing clinical trials employing them [78].	Cabozantinib
Ang/TIE2	Tumor vasculature reduction [136] and enhanced tumor sensitivity to antigen-specific cytotoxic T lymphocytes killing [136].	Multikinase inhibitor regorafenib FDA approved for the treatment of patients with metastatic CRC whose disease has progressed after prior therapy [126]. Phase I clinical trials with Trebaninib and Vanucizumab have showed no satisfactory results [137] or partial responses [138], respectively.	Cabozantinib, Ponatinib, Vandetanib
EPHR		Multikinase inhibitor regorafenib FDA approved for the treatment of patients with metastatic CRC whose disease has progressed after prior therapy [126].	Asatinib, Ponatinib, Vandetanib
RET	Vandetanib potently inhibits CRC cells proliferation and AKT and ERK phosphorylation [139].	Multikinase inhibitor regorafenib FDA approved for the treatment of patients with metastatic CRC whose disease has progressed after prior therapy [126].	Alectinib, Cabozantinib, Lenvatinib, Ponatenib, Sorafenib, Sunitinib, Vandetanib
DDR1	Strongly inhibits human CRC cell invasion and reduces their metastatic potential [140,141].	Promising pre-clinical studies [92,141].	Nilotinib
ALK/ROS	Inhibition of cell proliferation and MAPK/PI3K downregulation [142]	Clinical evidence supports that patient with advanced metastatic CRC harboring *ALK* fusions may benefit from targeted monotherapy with ALK inhibitors [142].	Alectinib, Brigatinib, Cabozantinib, Ceritinib, Crizotinib, Lorlatinib

Multi-kinase inhibitor targets and indications [143]. Afatinib (EGFR, ErbB2, ErbB4—NSCLC, squamous NSCLC), Alectinib (ALK, RET—NSCLC ALK+), Axitinib (VEGFR1/2/3, PDGFRb—RCC), Brigatinib (ALK, ROS1, IGF1R, FLT3, EGFR—NSCLC ALK+), Cabozantinib (RET, MET, VEGFR1/2/3, KIT, TRKB, FLT3, AXL, TIE2, ROS1—Metastatic medullary thyroid cancer, advanced RCC, HCC), Ceritinib (ALK, IGF1R, InsR, ROS1—NSCLC ALK+), Crizotinib (ALK, c-MET, ROS1—NSCLC ALK+, ROS1+ NSCLC), Dacomitinib (EGFR/ErbB2/ErbB4—EGFR- mutated NSCLC), Dasatinib (EGFR, KIT, EphA2, PDGFRb—Ph+ CML, ALL), Erlotinib (EGFR—NSCLC, pancreatic), Gefitinib (EGFR—NSCLC), Gilteritinib (FLT3—AML patients with FLT3 mutation), Imatinib (BCR-Abl, KIT, PDGFR—Ph+ CML or ALL, aggressive systemic mastocytosis, CEL, DFSP, HES, GIST, MDS/MDP), Lapatinib (EGFR, ErbB2—Breast), Larotrectinib (NTRK—solid tumors with *NTRK* fusions), Lenvatinib (VEGFR, FGFR, PDGFR, KIT, RET—DTC), Lorlatinib (ALK—ALK+ NSCLC), Midostaurin (FLT3—Acute myeloid leukemia with FLT3 mutation), Neratinib (ErbB2/HER2—Breast), Nilotinib (PDGFR, DDR1—Ph+ CML), Nintedanib (FGFR1/2/3, PDGFRa/b, VEGFR1/2/3, FTL3—Idiopatic pulmonary fibrosis), Osimertinib (EGFR—NSCLC), Pazopanib (VEGFR1/2/3, PDGFRa/b, FGFR1/3, KIT—RCC, soft tissue sarcoma), Ponatinib (VEGFR, PDGFR, FGFR, EphR, KIT, RET, TIE2, TLT3—Ph+ CML or ALL), Regorafenib (VEGFR1/2/3, KIT, PDGFRa/b, RET, FGFR1/2, TIE2, Eph2A—CRC, GIST), Sorafenib (RET, VEGFR1/2/3, PDGFRb—HCC, RCC, DTC), Sunitinib (PDGFRa/b, VEGFR1/2/3, KIT, FLT3, RET—RCC, GIST, PNET), Vandetanib (EGFR, VEGFR, RET, TIE2, EphR—Medullary thyroid cancer). Abbreviations: NSCLC: Non-Small-Cell Lung carcinoma: RCC: Renal cell carcinoma; HCC: Hepatocellular carcinoma; CML: Chronic myelogenous leukemia; CLL: Chronic lymphocytic leukemia; ALL: Acute lymphoblastic leukemia; AML: Adult acute myeloid leukemia; CEL: Chronic eosinophilic leukemia; DFSP: Dermatofibrosarcoma protuberans; HES: hypereosinophilic síndrome; GIST: Gastrointestinal stromal tumor; MDS/MDP: myelodysplastic/myeloproliferative disease; DTC: Differentiated thyroid carcinoma.
